# Penicillin-Susceptible *Streptococcus pneumoniae* Meningitis in Adults: Does the Ceftriaxone Dosing Matter?

**DOI:** 10.3390/antibiotics12050878

**Published:** 2023-05-09

**Authors:** Samuel Raemy, Carlo Casanova, Rossella Baldan, Erin Barreto, Aaron J. Tande, Andrea Endimiani, Stephen L. Leib, Urs Fischer, Parham Sendi

**Affiliations:** 1 Institute for Infectious Diseases, University of Bern, 3001 Bern, Switzerland; 2 Department of Pharmacy, Mayo Clinic, Rochester, MN 55902, USA; 3 Division of Public Health, Infectious Diseases and Occupational Medicine, Department of Medicine, Mayo Clinic, Rochester, MN 55902, USA; 4 Department of Neurology, University Hospital Bern, University of Bern, 3010 Bern, Switzerland; 5 Department of Neurology, University Hospital Basel, University of Basel, 4001 Basel, Switzerland

**Keywords:** meningitis, *Streptococcus pneumoniae*, ceftriaxone, penicillin, cerebrospinal fluid

## Abstract

The recommended empiric ceftriaxone dosing regimen for acute bacterial meningitis in adults is 2 g every 12 h. After penicillin-susceptible *Streptococcus pneumoniae* is isolated as a causative microorganism, the ceftriaxone dose may be continued or reduced to a single dose of 2 g every 24 h, per institutional preference. There is no clear guidance that indicates the superiority of one regimen over the other. The objective of this study was to evaluate the susceptibility of *S. pneumoniae* in the cerebral spinal fluid (CSF) of patients with meningitis and the relationship between ceftriaxone dose and clinical outcomes. We identified 52 patients with *S. pneumoniae* meningitis with positive CSF cultures who were treated at the University Hospital, Bern, Switzerland, over a 19-year period. We collected clinical and microbiological data for evaluation. Broth microdilution and Etest methods were performed to test penicillin and ceftriaxone susceptibility. All isolates were susceptible to ceftriaxone. Ceftriaxone was empirically used in 50 patients, with a starting dosing regimen of 2 g every 24 h in 15 patients and 2 g every 12 h in 35 patients. In 32 patients started on a twice-daily regimen (91%), doses were reduced to once daily after a median of 1.5 (95% CI 1–2) days. The overall in-hospital mortality was 15.4% (n = 8), and 45.7% of patients reported at least one sequela of meningitis at the last follow-up (median 375, 95% CI 189–1585 days). We found no statistical difference in outcome between the 2 g every 24 h and the 2 g every 12 h ceftriaxone dosing regimens. A ceftriaxone total daily dose of 2 g may be associated with similar outcomes to a 4 g total daily dose, provided that the causative organism is highly susceptible to ceftriaxone. The persistence of neurological and infection sequelae at the last follow-up underscores the need for optimal treatment of these complex infections.

## 1. Introduction

Community-acquired bacterial meningitis remains a feared infectious disease. The most common causative microorganisms are *Streptococcus pneumoniae* and *Neisseria meningitidis*. *S. pneumoniae* is responsible for two-thirds of meningitis cases in Europe and the United States [1,2,3]. Despite advances in medical care and the availability of vaccines, mortality from pneumococcal meningitis still ranges from 16% to 37%, and neurological sequelae are estimated to occur in 30–50% of surviving patients [1,3,4,5,6,7,8].

The treatment of bacterial meningitis includes antibiotic therapy that should be bactericidal and achieve adequate cerebrospinal fluid (CSF) concentrations. The European Society of Clinical Microbiology and Infectious Diseases (ESCMID) study group for infections of the brain (ESGIB) recommends the use of empirical ceftriaxone at a dose of 2 g q12h or a single dose of 4 g q24h (or cefotaxime 2 g q4–6h), along with vancomycin at a dose of 10–20 mg/kg q8–12h, to cover penicillin-resistant strains of *S. pneumoniae* in adult patients [9]. After *S. pneumoniae* is isolated and the penicillin susceptibility of the causative strain is confirmed, antibiotic treatment should be streamlined to either penicillin or ceftriaxone (or ampicillin, amoxicillin, or cefotaxime) [9]. Among patients treated with ceftriaxone, there is heterogeneity in clinical practice regarding dosing for directed therapy. Some institutions continue with the empiric dose of 2 g q12h or a single dose of 4 g q24h, while others favor a reduction to 2 g once daily. The advocates of the former dosing scheme base their rationale on poor antibiotic penetration into the CSF [10], the severity of the disease itself, and not more adverse events associated with 2 g q12h in comparison to 2 g q24h. Advocates of the single dose 2 g q24h dosing regimen point to (i) the low penicillin and ceftriaxone minimal inhibitory concentrations (MICs) of *S. pneumoniae*, (ii) the high ceftriaxone serum concentrations, (iii) the higher CSF:serum ratio of antibiotics when the blood–barrier tissue is inflamed, (iv) the clinical judgment at the bedside prior to dose reduction, and (v) antimicrobial stewardship programs.

To address this clinical question, we identified a subset of patients among our *S. pneumoniae* cohort in whom the growth of the microorganism was proven in CSF. The two aims of this study were (i) to investigate the penicillin and ceftriaxone MICs of *S. pneumoniae* grown in CSF and obtained from adults with acute bacterial meningitis, and (ii) to review antibiotic ceftriaxone dosing, clinical course, and outcome in these patients. From these findings, we discuss the rationale for ceftriaxone dosing and ceftriaxone pharmacokinetics in CSF.

## 2. Results

Between 2000 and 2018, 56 isolates of *S. pneumoniae* from patients with bacterial meningitis and growth in the CSF were identified. Medical records were available in all cases. However, four cases were excluded because of too many missing predefined variables (i.e., >10%). Ultimately, 52 cases with microbiological and clinical data were included in the analysis.

### 2.1. Antibiotic Susceptibility Retesting of 52 S. pneumoniae Isolates Obtained from CSF

All 52 isolates were thawed and retested for the purpose of this study. The results are displayed in Supplementary Table S1.

In the broth microdilution (BMD) method, 47 (90%) of the isolates were susceptible to penicillin, and 5 (10%) were resistant, according to the categorization of the 2022 Clinical and Laboratory Standards Institute (CLSI) [11]. All isolates were susceptible to ceftriaxone. The MIC_50_ and MIC_90_ for penicillin were 0.03 mg/L and 0.06 mg/L, while those for ceftriaxone were 0.016 mg/L and 0.0625 mg/L, respectively. Most isolates had a penicillin MIC of 0.03 mg/L (n = 41, 79%) and a ceftriaxone MIC of 0.016 mg/L (n = 39, 75%). The MIC values of penicillin and ceftriaxone were strongly correlated with each other (Pearson r = 0.9, *p* < 0.0001).

Using the Etest method, 48 (92%) isolates were susceptible to penicillin and 4 (8%) were resistant. One of these four resistant isolates had a MIC of 0.064 mg/L. All isolates were susceptible to ceftriaxone. The MIC_50_ and MIC_90_ for penicillin were 0.008 mg/L and 0.032 mg/L, while those for ceftriaxone were 0.008 mg/L and 0.023 mg/L, respectively. Similar to the BMD method results, the MIC values of penicillin and ceftriaxone strongly correlated with each other in the Etest method (Pearson r = 0.9, *p* < 0.0001).

### 2.2. Clinical Data of 52 Patients with Pneumococcal Meningitis

Patient characteristics and previously described risk factors for bacterial meningitis are listed in Table 1. The majority of patients (n = 37 of 51 (72.6%) with available data for this variable) reported a respiratory or otogenic infection prior to the onset of meningitis symptoms. Almost 60% of patients had at least one comorbid condition. The three most common comorbidities were diabetes mellitus, impaired kidney function, and chronic heart disease.

Table 2 illustrates the clinical and laboratory findings of the 52 included patients. The classic triad of bacterial meningitis (altered mental status, neck stiffness, and fever) was present in 60% of cases. The duration of meningitis symptoms prior to presentation was short (median 1 day, 95% CI 0.5–3 days), illustrating the acute characteristics of the disease. The median C-reactive protein (CRP) level at the time of admission was 168.5 mg/L (n = 50, 95% CI 99–260 mg/L), and after 24 h it was 263 mg/L (n = 48, 95% CI 215–331 mg/L). CSF findings were consistent with those described for acute bacterial meningitis (i.e., elevated cell count and protein levels, low glucose levels) [9].

### 2.3. Evidence of S. pneumoniae in Samples Other Than CSF

Blood cultures were positive for *S. pneumoniae* in 34 of 46 (73.9%) patients in whom blood cultures were obtained. A urine antigen test for *S. pneumoniae* was performed in 14 (26.9%) patients and was positive in 12 (85.7%) of them.

### 2.4. Antibiotic Treatment with a Specific Focus on Ceftriaxone Dosing

Overall, as frequently observed in clinical practice, the antibiotic treatment regimens were heterogeneous among the 52 patients. In five (9.6%) cases, another infection focus was first suspected (e.g., respiratory tract infection) prior to the suspicion or diagnosis of bacterial meningitis and switching to the appropriate antibiotic treatment regimen. In these cases, the initial empiric treatment consisted of amoxicillin/clavulanate (four cases) and clindamycin (one case). In 50 of 52 patients, empirical treatment for bacterial meningitis consisted of ceftriaxone plus vancomycin with or without amoxicillin. In two cases, another antibiotic compound was chosen for empiric therapy. In 35 (70%) of these 50 patients, the prescribed starting dose was 2 g q12h, and in 15 (30%) it was 2 g q24h. One of the 35 patients with a prescribed dose of 2 g q12h died within 6 h after admission. In 32 of the other 34 patients, the dose was reduced to 2 g q24h after a median treatment duration of 1.5 (95% CI 1–2) days. Hence, the antibiotic treatment in 47 (32 + 15) of 50 patients—in whom ceftriaxone was initiated empirically—was continued with 2 g ceftriaxone q24h for a median duration of 8 days. In 10 of these patients, treatment was eventually switched to penicillin G. Only in 3 (6%) of the 50 patients was ceftriaxone continued at the empiric dose of 2 g q12h for a median duration of 15 days.

### 2.5. Outcome

We differentiated between in-hospital outcomes and outcomes at the latest follow-up. The primary outcome (in-hospital death) occurred in eight (15.4%) patients. The median age of these eight patients was 75 (range 65–80) years. In-hospital complications of bacterial meningitis occurred in nearly 65% of the cases. Seizures, elevated intracranial pressure, cerebral ischemia, and sinus venous thrombosis were the most common complications (Table 3). One-third of patients indicated impaired hearing at the time of hospital discharge.

A total of 9 of the 44 discharged patients were lost to follow-up, and the median follow-up of the remaining 35 patients was 375 days (95% CI 189–1585 days). Nearly half (45.7%) of these patients indicated at least one sequela from pneumococcal meningitis. The most frequently reported sequelae included headaches, vestibulopathy, and impaired hearing (Table 3).

### 2.6. Association of Outcome, MIC of S. pneumoniae, and Ceftriaxone Dosing

Among the eight patients who died during hospitalization, all but one were initially treated with 2 g q12h (Table 4). One patient died within 6 h after admission and received only the first dose (no. 19). One patient receiving 2 g q24h died after 5 days (no. 7). Among patients who died, the *S. pneumoniae* isolates were consistently susceptible to ceftriaxone and penicillin via Etest. One isolate was categorized as resistant to penicillin only in the BMD test (no. 7).

The recommended dosing scheme for amoxicillin was 2 g q4h and for vancomycin, 15 mg/kg body weight q12h, provided there was no impairment of renal function. In this table, “ceftriaxone (4 g/24 h)” corresponds to a dosing regimen of 2 g q12h.

Considering that only one out of eight patients who died during hospitalization was initially treated with the lower ceftriaxone dose (2 g q12h), we focused in the next step on the association of outcome at the latest follow-up and initial ceftriaxone dosing. For this analysis, we excluded patients lost to follow-up (n = 8) and one additional patient who was lost to follow-up and in whom the initial antibiotic compound was not ceftriaxone. We found no statistical difference in neurological impairment at discharge or in sequelae when comparing patients who were initially treated with ceftriaxone 2 g q12h or 2 g q24h (Table 5).

## 3. Discussion

In this study, we aimed to explore the relationship between ceftriaxone dosing, MICs of *S. pneumoniae*, and clinical outcomes in cases of adult bacterial meningitis. Because the penetration and activity of antibiotics in the CSF are frequently raised as arguments for increased antibiotic dosing, we selected only cases with proven culture growth of *S. pneumoniae* in the CSF.

We identified 56 *S. pneumoniae* isolates over 19 years (mean 2.9/year). We were unable to provide precise proportions of pneumococcal meningitis cases with and without bacterial growth in CSF because our *S. pneumoniae* cohort differentiated only between invasive and noninvasive isolates [12]. For comparison, in the last decade, between 120 and 160 invasive pneumococcal diseases per year were noted in the Canton of Bern [13]. It has been estimated that meningitis occurs in approximately 4% of these cases [14]. After extrapolating this proportion to our pneumococcal cohort, an estimated five to six proven pneumococcal meningitis cases per year in our region is plausible. Thus, it is reasonable to further estimate that in approximately 50% of those cases, evidence of bacterial growth in the CSF is detected. This is noteworthy, considering that intravenous antibiotics are administered prior to lumbar puncture.

Depending on the chosen test method, 90.4% of *S. pneumoniae* isolates were susceptible to penicillin with the BMD and 92% with the Etest method. Interpreting a penicillin MIC value of 0.064 mg/L in the Etest is challenging as it falls between the lower and upper values imprinted on the stripes, which are 0.047 mg/L and 0.094 mg/L, respectively. A value of 0.064 mg/L in the Etest was observed in only one isolate in this study. All 52 isolates in this study were susceptible to ceftriaxone. A previous study from the Swiss National Reference Centre for Pneumococci reported 9.8% penicillin non-susceptible strains and 2.2% ceftriaxone non-susceptible strains among 10,996 invasive isolates collected from 2004 to 2014 [12]. The penicillin and ceftriaxone susceptibility breakpoints for pneumococcal meningitis were adapted in 2008. Our collection included isolates from 2000 to 2018, and CLSI breakpoint recommendations for 2022 were used [11]. However, even when we used the pre-2008 breakpoints, this did not significantly affect the proportion range in our susceptibility test results.

The presentation, clinical and laboratory findings, and outcomes of our study are in line with those of other studies [9]. The triad of fever, neck stiffness, and altered mental status was present in 60% of cases and was higher than that reported elsewhere (ranging from 41% to 51%) [9]. A possible reason for this finding is that we included only those cases with bacterial growth in the CSF, i.e., a subpopulation with advanced disease. The mortality rate from pneumococcal meningitis still ranges from 16% to 37%, and neurological sequelae are estimated to occur in 30–50% of surviving adults [15,16]. In line with these results, the mortality rate in our study, which included only patients with growth in the CSF, was 15.4%, and 45.7% of patients reported at least one sequelae symptom.

ESCMID and UK guidelines recommend the use of third-generation cephalosporins alone only when the prevalence proportion of cephalosporin-resistant isolates is <1% [9,17]. Considering that all isolates were ceftriaxone-susceptible in our study, this recommendation fits with our institutional practice. Nonetheless, all patients but one received empiric combination therapy that included ceftriaxone and vancomycin. Of note, the proportion of ceftriaxone non-susceptible *S. pneumoniae* isolates—when analyzing all infection sites from 2004 to 2014—was 2.2% [12]. Our institutional practice to down dose ceftriaxone from 2 g q12h to 2 g q24h in penicillin-susceptible pneumococcus cases is based on the low MIC values observed in the Swiss National Reference Centre for Pneumococci and the following postulated pharmacological properties of ceftriaxone.

In healthy adults, the maximum plasma ceftriaxone concentration (C_max_ mean ± standard deviation [SD]) after 2 g q24h at day 1 is 239 (SD ± 26) mg/L and at day 4 is 260 (SD ± 24) mg/L, and the minimum concentration (C_min_, mean) at day 1 is 13 (SD ± 5.5) mg/L and at day 4 is 15 (SD ± 6.2) mg/L [18]. The elimination half-life is 6.2 (range 5.3–7.9) h, and the binding to plasma proteins is 90–95% [18,19]. The binding proportion decreases with increased ceftriaxone plasma concentrations [19,20]. However, patients with acute bacterial meningitis are typically critically ill, and their physiological condition affects the pharmacodynamic and kinetic parameters of ceftriaxone (e.g., hypo-albuminemia) [21]. Both elimination and antimicrobial activity are limited to the unbound fraction of ceftriaxone.

Schleibinger et al. [22] investigated 69 plasma samples from 20 intensive care unit patients treated with ceftriaxone 2 g q24h. In this population, the elimination half-life was 14.5 h and the median unbound fraction was 33.0% (interquartile range 20.2–44.5%) [22]. A higher unbound fraction increases the pharmacologically active antibiotic available for antibacterial activity (ƒT_>MIC_) [23]. In Schleibinger et al.’s study [22], in all patients, unbound concentrations during treatment with ceftriaxone 2 g q24h remained above the MIC of ≤1 mg/L throughout the whole dosing interval. Heffernan et al. [21] investigated 474 samples from 36 critically ill patients. The authors calculated the probability of target attainment for an unbound ceftriaxone concentration (C_min_/MIC of >1) over the first 24 h of therapy based on plasma albumin concentrations and renal clearance. Of note, the elimination of the unbound fraction may also be enhanced with augmented renal clearance [21,22]. For organisms with a MIC of ≤0.25 mg/L, the probability was 88% when assuming a creatinine clearance of 160 mL/min and plasma albumin of 20 g/L. The probability was 95–100% for all other possible constellations of these two parameters [21]. For comparison, the MIC_90_ for ceftriaxone among the *S. pneumoniae* isolates investigated in this study was 0.0625 mg/L (BMD) and 0.023 (Etest).

High (unbound) plasma concentrations of ceftriaxone led to higher absolute concentrations beyond the blood–brain barrier. For the treatment of meningitis, however, more than the aforementioned pharmacokinetic and dynamic parameters have to be taken into consideration. The penetration of ceftriaxone into the CSF in the absence of meningeal inflammation is relatively poor. Studies reported a CSF/plasma ratio of 1.5% for ceftriaxone [24] or an AUC_CSF_/AUC_serum_ of 0.7% [10]. Within the CSF compartment, ceftriaxone is not metabolized [25]. Moreover, there is no affinity for ceftriaxone for drug efflux pumps at the blood–brain or blood–CSF barrier [26]. Thus, the elimination of ceftriaxone from CSF is slow. It occurs via bulk flow and retrograde diffusion over the blood–brain barrier [10]. The estimated elimination half-life of the CSF is 15.7–18.4 h [20].

Conversely, the penetration of ceftriaxone into the CSF is increased in bacterial meningitis because the inflammatory cascade leads to strong alterations of the blood–brain barrier [1,20]. In addition, ceftriaxone follows the concentration gradient into the CSF via paracellular diffusion. Prásil et al. [27] described a CSF/serum ratio of ≥10% in patients with high CRP and fibrinogen serum levels, as well as a high neutrophil count in the CSF. Grégoire et al. [28] reported a median value of the CSF/plasma total ceftriaxone ratio of 14.39% (range 5.86–65.94%) in eight patients with meningitis. Other studies have reported absolute values of CSF ceftriaxone total concentrations. In a subgroup of four patients with pneumococcal meningitis from two French cohorts, the reported total concentrations were 9.7, 24.0, 27.1, and 91.2 mg/L [29]. Studies from South Africa and Turkey reported total ceftriaxone concentrations in CSF in a steady state ranging from 3 to 5 mg/L [30,31]. Of note, concomitant dexamethasone use does not seem to significantly affect ceftriaxone penetration into the CSF in adult patients with acute bacterial meningitis [31]. The ceftriaxone dosing in the aforementioned studies was 2g q12h, 4g q24h, or even higher [27,28,29,30,31]. The time interval between the administration of ceftriaxone and the harvesting of CSF for drug concentration analysis varied between these studies, making comparability of the results difficult. Although the permeability of the blood–brain barrier for compounds is increased during the acute phase of bacterial meningitis, it returns to previous levels with poor penetration of antibiotics into the CSF when patients recover from the infection [24].

Taken together, these results show that in acute bacterial meningitis, the unbound proportion of ceftriaxone increases with increasing total plasma concentration and in critically ill patients with hypoalbuminemia. Furthermore, ceftriaxone penetrates 10 times as much or more into the CSF in the presence of meningeal inflammation as it does in a condition without meningeal inflammation. The absolute ceftriaxone concentration achieved in the CSF is from 100 to 1000 times or more than that of the MICs of penicillin-susceptible pneumococci. Elimination from the CSF is relatively slow; hence, the half-time in this compartment is considerably prolonged.

To our knowledge, no study has systematically investigated the outcome of pneumococcal meningitis with a ceftriaxone dose reduction strategy when the causative strain is susceptible. A Monte Carlo simulation estimated the probability of target attainment for total drug CSF concentrations at 50% and 100% *t* > MIC for ceftriaxone 2 g q12h [32]. The probability of achieving 50% *t* > MIC in the CSF approached ≥90% only when the ceftriaxone MIC was ≤0.03 mg/L. The implications of pharmacodynamic studies need to be reexamined in the clinical setting, as certain assumptions made in such studies could be questioned.

Our study has limitations. No semiquantitative or quantitative method was applied to evaluate the growth or concentrations of microorganisms in CSF, and no ceftriaxone drug concentrations were measured in plasma or CSF in these cases. Hence, we were unable to perform pharmacometric studies. The *t* > MIC estimates were calculated from theoretical extrapolations of ceftriaxone pharmacokinetic and dynamic studies, as well as from our MIC susceptibility testing results. Because of the retrospective nature of the study, we were unable to reconstruct the rationale for why the initial empiric dose was ‘only’ 2 g q24h in 15 cases or why 2 g q12h was continued in 3 cases despite the fact that the causative microorganism was highly susceptible. Similarly, we were unable to evaluate other factors that may have influenced mortality (e.g., age, year of death). The uneven distribution of the dosing groups and relatively low sample size did not allow for firm statistical conclusions. However, the clinical outcome results of our study are comparable to those of others, indicating that the reducing the ceftriaxone dose after susceptibility test results are available does not necessarily lead to a poorer outcome. The early recognition of signs of meningitis and the rapid administration of antibiotics are still pivotal to the prognosis.

## 4. Materials and Methods

Clinical pneumococcal isolates from normally sterile body sites (blood, CSF, joint, pleural, and peritoneal fluid) have been collected in our institution since 2000. For this study, we included only *S. pneumoniae* strains with proven culture growth in CSF obtained from adult patients who were referred or treated for bacterial meningitis at the University Hospital of Bern (Inselspital), Bern, Switzerland. The rationale for this selection is that we could evaluate the clinical outcome of pneumococcal meningitis in association with ceftriaxone dosing and include the embedded discussion on MICs and antibiotic penetration into the CSF. We included isolates obtained from 2000 to 2018. The rationale for this time span is that it included the publication year of the ESCMID guidelines for bacterial meningitis [9] and a clinical practice adaptation period of 2 years.

### 4.1. Antibiotic Susceptibility Testing

All isolates were identified according to the defined protocol of the Swiss National Center for Pneumococci [12]. Isolates were tested for penicillin and ceftriaxone susceptibility by using two different methods: the BMD method with cation-adjusted Mueller-Hinton broth supplemented with 5% lysed horse blood and the Etest method (penicillin from bioMérieux SA, Marcy-l’Étoile, France; ceftriaxone from Liofilchem, Roseto degli Abruzzi, TE, Italy) on MHA with 5% mechanically defibrinated horse blood and 20 μg/mL nicotinamide adenine dinucleotide [11]. The following CLSI categorization from 2022 was used for *S. pneumoniae* and meningitis [11]: penicillin ≤ 0.06 mg/L = susceptible, ≥0.12 mg/L = resistant; ceftriaxone ≤ 0.5 mg/L = susceptible, ≥2 mg/L = resistant.

### 4.2. Clinical Data

To evaluate the association between microbiological and clinical data, we reviewed patient charts corresponding to the *S. pneumoniae* isolates. Only adult patients (≥18 years of age) were included. Access to charts was available only for patients treated at the University Hospital Bern (Inselspital), Bern, Switzerland. The following variables were obtained: age, gender, comorbidities, diabetes mellitus, impaired renal function, chronic heart disease, immunosuppression, chronic alcohol abuse, evidence of a recent respiratory infection, and travel history. In addition, symptoms and findings were obtained, as well as laboratory parameters at clinical presentation.

Treatment analysis included antibiotic therapy (agent, dosing, and time of administration) in comparison to the outcome. The primary outcome was defined as in-hospital death. Secondary outcomes included in-hospital complications (all causes), neurological impairment at discharge, and sequelae at the latest follow-up. The maximum follow-up was defined as the latest available contact at the University Hospital Bern (Inselspital), irrespective of the cause of contact.

### 4.3. Statistical Analysis

For all variables, the number and proportion of available results were obtained. Patients with more than 10% missing variables were excluded from the clinical analysis. Continuous variables were registered as mean and SD. Categorical variables were registered as counts, and groups were compared via the chi-square test and Fisher’s exact test. *p*-values of <0.05 were interpreted as being statistically significant. GraphPad Prism was used for statistical analysis and the graphical depiction of the obtained data.

## 5. Conclusions

In our cohort of *S. pneumoniae* isolates obtained from CSF, susceptibility to penicillin and ceftriaxone was high, and the ceftriaxone MIC values were low. The vast majority of patients from whom these isolates were obtained were eventually treated with ceftriaxone at a dose of 2 g q24h. Mortality was 15.4%, and 45.7% of patients reported at least one sequela in the follow-up examination, indicating that acute pneumococcal meningitis is a severe disease. However, these results are consistent with those reported by others. Our study indicated that the ceftriaxone dosing regimens of 2 g q24h or 2 g q12h may have a similar outcome, provided that the causative microorganism is highly susceptible to ceftriaxone. Thus, reducing the ceftriaxone dose from 4 g daily (empiric therapy) to 2 g daily (targeted therapy) after susceptibility results are available may not influence the outcome.

## Figures and Tables

**Table 1:** Patient characteristics.

Characteristics	N or Median (% or 95% CI)
Male	23 (44.2%)
Female	29 (55.8%)
**Age** (years)	57.5 (95% CI 52–75)
**Comorbidities present** ^3^	30 (58.8%)
Diabetes mellitus (Type 1 or 2)	2 (3.9%)
Impaired kidney function ^1,3^	8 (15.7%)
Chronic heart disease	28 (53.9%)
**Any immunocompromising factors**	9 (17.3%)
HIV	3 (5.8%)
Use of systemic corticosteroids > 14 days	4 (7.7%)
Other immunocompromising factors ^2^	2 (3.9%)

^1^ Defined as chronic kidney disease stage 3A or below (i.e., estimated glomerular filtration rate 45–59 mL/min or below). ^2^ Including splenectomy, humoral immunodeficiency, and hepatitis C. ^3^ Denominators = 51.

**Table 2:** Clinical findings and results of CSF fluid analysis.

Findings at Presentation to ED	N or Median (% or 95% CI)
**Typical meningitis symptoms**	
Altered mental status (GCS < 15)	48 (92.3%)
Neck stiffness	38 (84.4%)
Fever	33 (64.7%)
All three findings (meningitis triad)	27 (60.0%)
**Other symptoms**	
Headaches	37 (88.1%)
Vomiting	18 (36.7%)
Unspecific symptoms ^1^	13 (25.5%)
**Duration of symptoms prior to admission (days)**	1 (95% CI 0.5–3)
**Cerebrospinal fluid analysis**	
WBC count in cells/μL (reference value < 5) Glucose in mmol/L (reference 2.5–4.4) Protein in mg/L (reference 20–40)	760 (95% CI 357–1800) 1.4 (95% CI 0.07–2.63) 500.0 (95% CI 360–595)

ED, emergency department; GCS, Glasgow Coma Scale; WBC, white blood cell. ^1^ Including symptoms of upper respiratory tract infection, diarrhea, ear pain, symptoms of lower respiratory tract infection, thoracic pain, and fatigue/feeling unwell.

**Table 3:** Outcomes of pneumococcal meningitis in 52 patients with bacterial growth in the CSF.

Outcome	N or Median (% or 95% CI)
**Primary outcome**	
In-hospital death	8 (15.4%)
**Secondary outcomes ^1^**	
Seizures	12 (23.5%)
ICP↑	8 (15.7%)
Cerebral ischemia	5 (9.8%)
Sinus venous thrombosis	4 (7.8%)
**Neurological impairment at discharge ^2^**	
Impairment of hearing	14 (33.3%)
Tympanostomy tube	15 (30.0%)
**Sequelae at last follow-up ^3^**	16 (45.7%)
Median follow-up in days	375, 95% CI 189–1585
Frequent headaches	6 (17.1%)
Vestibulopathy	6 (17.1%)
Impaired hearing	6 (17.1%)
Low-grade neurocognitive impairment ^4^	3 (8.6%)
Postinfectious hydrocephalus	2 (5.7%)
Sensory neuropathy	1 (2.9%)
Loss of smell and taste sense	1 (2.9%)
Epilepsy	1 (2.9%)

ICP↑, intracranial pressure elevated. ^1^ Denominator = 51; ^2^ denominator = 42, ^3^ denominator = 35. ^4^ Including memory deficits, emotional instability, inability to focus mentally, and fatigue.

**Table 4:** Patients with the outcome of in-hospital death, antibiotic treatment regimens, and susceptibility testing results.

Patient No.	Age	Sex	No. Days of Antibiotic Treatment	Antibiotic Treatment Regimen	PEN MIC mg/L	CRO MIC mg/L	Inhospital Death
3	77	F	12	Day 1: ceftriaxone (4 g/24 h) + vancomycin Day 2: ceftriaxone (2 g/24 h) Days 2–12: penicillin G 6 × 4 Mio IE/24 h	BMD 0.03 Etest 0.006	BMD 0.016 Etest 0.004	21
7	80	M	5	Days 1–5: ceftriaxone (2 g/24 h) Day 2: ceftriaxone + vancomycin + amoxicillin	BMD 0.12 Etest 0.047	BMD 0.0625 Etest 0.016	5
12	65	M	13	Day 1: ceftriaxone (4 g/24 h) + amoxicillin Days 2–3: ceftriaxone (4 g/24 h) Days 4–8: ceftriaxone (2 g/24 h) + pip/tazo Days 9–13: vancomycin + cefepime	BMD 0.03 Etest 0.012	BMD 0.0625 Etest 0.03125	13
18	65	F	13	Days 1–3: ceftriaxone (4 g/24 h) + vancomycin Days 4–11: ceftriaxone (2 g/24 h)	BMD 0.03 Etest 0.012	BMD 0.016 Etest 0.008	13
19	81	F	1	Day 1: ceftriaxone (2 g)	BMD 0.03 Etest 0.006	BMD 0.016 Etest 0.006	1 (6 h)
22	86	F	3	Days 1–3: ceftriaxone (4 g/24 h) + vancomycin + amoxicillin	BMD 0.03 Etest 0.016	BMD 0.03125 Etest 0.012	3
25	66	M	37	Day 1: ceftriaxone (4 g/24 h) + vancomycin Days 2–4: ceftriaxone (2 g/24 h) + vancomycin Days 5–10: penicillin G 6 × 4 Mio IE/24 h Days 15–37: cefepime + vancomycin + Metronidazole	BMD 0.03 Etest 0.008	BMD 0.016 Etest 0.008	38
46	73	F	3	Days 1–3: ceftriaxone (4 g/24 h) + amoxicillin	BMD 0.03 Etest 0.008	BMD 0.016 Etest 0.004	3

No., number; PEN, penicillin; CRO, ceftriaxone; BMD, broth microdilution; pip/tazo, piperacillin/tazobactam; M, male; F, female.

**Table 5:** Comparisons of neurological impairment at discharge or of sequelae in patients who were initially treated with ceftriaxone 2 g q12h or 2 g q24h.

Outcome/Initial Ceftriaxone Dose	No. Patients	2 g q12h N (%)	2 g q24h N (%)	*p* Value
**No. of patients at discharge**	**42**	**28**	**14**	-
**Any neurological impairment at discharge**	23	16 (57.1)	7 (50.0)	0.661
Impairment of hearing at discharge	14	10 (35.7)	4 (28.6)	0.643
**No. of patients with follow-up**	**34**	**22**	**12**	-
**Any sequelae at the last follow-up**	15	11 (50.0)	4 (33.3)	0.349
- Frequent headaches	6	5 (17.9)	1 (7.1)	0.292
- Vestibulopathy	6	3 (10.7)	3 (21.4)	0.406
- Impaired hearing	6	4 (14.3)	2 (14.3)	0.911
- Mild neurocognitive impairment	3	2 (7.1)	1 (7.1)	0.940
- Postinfectious hydrocephalus	2	2 (7.1)	0 (0)	0.886

## Data Availability

The data presented in this study are available on request from the corresponding author. The data are not publicly available due to concerns about privacy and ethical aspects.

## Supplementary Materials

The following supporting information can be downloaded at: https://www.mdpi.com/article/10.3390/antibiotics12050878/s1, Table S1: MIC values of penicillin and ceftriaxone.
