# Supplementary material for: Penicillin-Susceptible Streptococcus pneumoniae Meningitis in Adults: Does the Ceftriaxone Dosing Matter?
Source: Antibiotics (Basel). 2023 May 9;12(5):878. doi: 10.3390/antibiotics12050878 (PMC10215215; doi:10.3390/antibiotics12050878)
Supplement: Supplementary file 1 [file antibiotics-12-00878-s001.zip › antibiotics-2388623-supplementary.pdf]

antibiotics

MDPI

Supplementary Materials

Penicillin-Susceptible *Streptococcus pneumoniae* Meningitis in Adults: Does the Ceftriaxone Dosing Matter?

Samuel Raemy [1], Carlo Casanova [1], Rossella Baldan [1], Erin Barreto [2], Aaron J. Tande [3], Andrea Endimiani [1], Stephen L. Leib [1], Urs Fischer [4,5] and Parham Sendi [1,*]

1 Institute for Infectious Diseases, University of Bern, 3001 Bern, Switzerland
2 Department of Pharmacy, Mayo Clinic, Rochester, MN 55902, USA
3 Division of Public Health, Infectious Diseases and Occupational Medicine, Department of Medicine, Mayo Clinic, Rochester, MN 55902, USA
4 Department of Neurology, University Hospital Bern, University of Bern, 3010 Bern, Switzerland
5 Department of Neurology, University Hospital Basel, University of Basel, 4001 Basel, Switzerland
* Correspondence: parham.sendi@unibe.ch

Supplementary Table S1: MIC values of penicillin and ceftriaxone of 52 included isolates.

		**Penicillin (mg/L)**		**Ceftriaxone (mg/L)**	
	Pat. ID	E-Test	BMD	ETest	BMD
1	43	0.008	0.03	0.003	0.016
2	51	0.003	0.03	0.004	0.016
3	5	0.004	0.03	0.004	0.016
4	3	0.006	0.03	0.004	0.016
5	41	0.006	0.03	0.004	0.016
6	24	0.006	0.03	0.004	0.016
7	46	0.008	0.03	0.004	0.016
8	40	0.008	0.03	0.004	0.016
9	34	0.008	0.03	0.004	0.016
10	28	0.008	0.03	0.004	0.016
11	4	0.008	0.0075	0.006	0.016
12	19	0.006	0.03	0.006	0.016
13	56	0.008	0.03	0.006	0.016
14	49	0.008	0.03	0.006	0.016
15	48	0.008	0.03	0.006	0.016
16	33	0.008	0.03	0.006	0.016
17	30	0.008	0.03	0.006	0.016
18	29	0.008	0.03	0.006	0.016
19	15	0.008	0.03	0.006	0.016
20	47	0.012	0.03	0.006	0.016
21	45	0.012	0.03	0.006	0.016
22	38	0.012	0.03	0.006	0.016
23	20	0.008	0.01	0.008	0.016
24	37	0.008	0.03	0.008	0.016
25	27	0.008	0.03	0.008	0.016
26	25	0.008	0.03	0.008	0.016
27	17	0.008	0.03	0.008	0.016

28	42	0.012	0.03	0.008	0.016
29	39	0.012	0.03	0.008	0.016
30	32	0.012	0.03	0.008	0.016
31	18	0.012	0.03	0.008	0.016
32	52	0.016	0.03	0.008	0.016
33	21	0.016	0.03	0.008	0.016
34	36	0.012	0.03	0.012	0.016
35	23	0.012	0.03	0.012	0.016
36	1	0.016	0.03	0.012	0.016
37	35	0.016	0.03	0.012	0.016
38	13	0.016	0.03	0.012	0.016
39	50	0.006	0.03	0.006	0.03125
40	2	0.008	0.03	0.008	0.03125
41	10	0.008	0.03	0.008	0.03125
42	12	0.012	0.03	0.012	0.03125
43	22	0.016	0.03	0.012	0.03125
44	31	0.064	0.12	0.023	0.062
45	16	0.012	0.06	0.008	0.0625
46	14	0.016	0.06	0.016	0.0625
47	7	0.047	0.12	0.016	0.0625
48	44	0.016	0.06	0.032	0.0625
49	11	0.032	0.06	0.032	0.125
50	8	0.094	0.24	0.032	0.125
51	6	0.19	0.48	0.094	0.25
52	9	0.38	0.48	0.38	0.5

Abbreviation: BMD, broth microdilution; The sorting of values is in the following order (from lowest to highest value): BMD ceftriaxone, Etest ceftriaxone, BMD penicillin, Etest, penicillin. The following CLSI categorization from 2022 was used for *S. pneumoniae* and meningitis: penicillin ≤ 0.06 mg/L = susceptible, ≥ 0.12 mg/L = resistant; ceftriaxone ≤ 0.5 mg/L = susceptible, ≥ 2 mg/L = resistant. MIC values in the red boxes indicate penicillin-resistant strains.

Clinical and Laboratory Standards Institute (CLSI 2022). Supplement M100. Performance standards for antimicrobial susceptibility testing. 32nd edition, 2022, Table 2G.
